# Technological Aspects of the Production of Fructo and Galacto-Oligosaccharides. Enzymatic Synthesis and Hydrolysis

**DOI:** 10.3389/fnut.2019.00078

**Published:** 2019-05-31

**Authors:** Gonçalo N. Martins, Maria Micaela Ureta, E. Elizabeth Tymczyszyn, Paula C. Castilho, Andrea Gomez-Zavaglia

**Affiliations:** ^1^Centro de Química da Madeira, Universidade da Madeira, Campus da Penteada, Funchal, Portugal; ^2^Center for Research and Development in Food Cryotechnology (CIDCA, CCT-CONICET La Plata), La Plata, Argentina; ^3^Laboratorio de Microbiología Molecular, Departamento de Ciencia y Tecnología, Universidad Nacional de Quilmes, Bernal, Argentina

**Keywords:** fructo-oligosaccharides, galacto-oligosaccharides, enzymatic synthesis, hydrolysis, properties and applications, alternative substrates

## Abstract

Fructo- and galacto-oligosaccharides (FOS and GOS) are non-digestible oligosaccharides with prebiotic properties that can be incorporated into a wide number of products. This review details the general outlines for the production of FOS and GOS, both by enzymatic synthesis using disaccharides or other substrates, and by hydrolysis of polysaccharides. Special emphasis is laid on technological aspects, raw materials, properties, and applications.

## Introduction

The first reference to prebiotic concept dates from 1954, when Gyorgy reported that a component of human milk (N-acetyl-glucosamine) promoted the growth of a strain from the genus *Bifidobacterium*. A few years later, Petuely (1) recognized lactulose as a *bifidus factor*. Almost 20 years after, Japanese researchers reported that several non-digestible oligosaccharides were *bifidus factors* (2, 3). The term prebiotic as such, was defined in 1995 (4), as “non-digestible food components that beneficially affect the host by selectively stimulating the growth and/or activity of one or a limited number of bacteria in the colon, thus improving host health” (5).

Since then, the original definition was subjected to several revisions. According to the most recent one, prebiotics are “substrates that are selectively used by host microorganisms conferring a health benefit” (6). Research in different domains (glycomics, proteomics, etc.), reveals more complex interactions of putative prebiotics with the host, thus this definition is far from being the last one. From a scientific point of view, it is a subject still under development and the advances in this issue impact not only on the scientific community, but also on regulatory agencies, food industries, consumers and healthcare professionals (7).

Regardless the definition, fructo- and galacto-oligosaccharides (FOS and GOS) are widely known because of their prebiotic properties. Additionally, their nutritional properties are also important, they are low caloric sweeteners, give a feeling of satiety, contribute to body weight control, relieve constipation, have a low glycemic index and are not cariogenic (8). GOS and FOS are used in the formulation of dairy products, different types of beverages, bakery products, and some sweets, converting them in functional foods (9). Moreover, they are extensively employed in infant formula to stimulate the development of newborn microbiota (10, 11).

As GOS and FOS can be incorporated in many products, their demand has exponentially increased worldwide over time (12). Japan has been pioneer in the production and consumption of FOS and GOS. It was the first country to incorporate non-digestible oligosaccharides in foods, being a world leader in the use of prebiotics as functional ingredients.

In 2006 the functional food market was estimated to be $20 billion in the United States, $15 billion in Europe, and $12 billion in Japan, growing at an annual rate of 7.5% (13). Particularly the prebiotic market reached $200 million in 2015, with an increase rate of about 15% per year (www.reuters.com/article/pressRelease). What is more, according to Global Market Insights, INC (Delaware, USA), the global prebiotic market is expected to surpass $8.5 billion by 2024 (14). It is remarkable that the increase of the prebiotic market is much higher than that of the food market as a whole, whose increase is about 2% per year.

Considering the economical and nutritional importance of FOS and GOS, this review will be focused on their obtaining. From a technological point of view, these prebiotics can be produced either from natural sources or by enzymatic synthesis using disaccharides or other substrates as raw materials. Furthermore, the hydrolysis of polysaccharides present in many fruits and vegetables is another way for obtaining FOS and GOS. Different methods for producing FOS and GOS will be presented, with special emphasis on raw materials, suitable for both synthesis and hydrolysis reactions. Additional properties and applications of FOS and GOS will be also discussed.

## FOS

Fructo-oligosaccharides (FOS) are composed of a small number of fructose units linked by (2→ 1)-β-glycosidic bonds and having a single D-glucosyl unit at the non-reducing end. Particularly, short chain FOS are mixtures of the smallest oligosaccharides, namely 1-kestose [degree of polymerization (DP) equal to 3], nystose (DP4) and 1^F^-fructofuranosylnystose (DP5) (4). They can be obtained either by enzymatic synthesis or by hydrolysis of inulin from natural sources mainly from roots of chicory, artichoke, yacon, dahlia or agave. This later method leads to higher molecular weight FOS.

### FOS Obtained by Enzymatic Synthesis

The production of FOS obtained by enzymatic synthesis involves transfructosylation reactions where fructosyltransferases (β-fructofuranosidase, EC 3.2.1.26 or β-D-fructosyltransferase, EC 2.4.1.9) act as biocatalysts (10, 11, 15–17). Meiji Seika Kaisha Ltd. pioneered the production of FOS by enzymatic synthesis using the organism of *Aspergillus niger*. Nowadays, this company is one of the leaders of short chain FOS market all over the world, their products are labeled under the brand names Actilight® in Europe and Meioligo in Asia (18). Additionally, NutraFlora® from Ingredion group companies is another brand of short chain FOS that leaders the market in North and South America and Australia (19).

**Figure 1:**
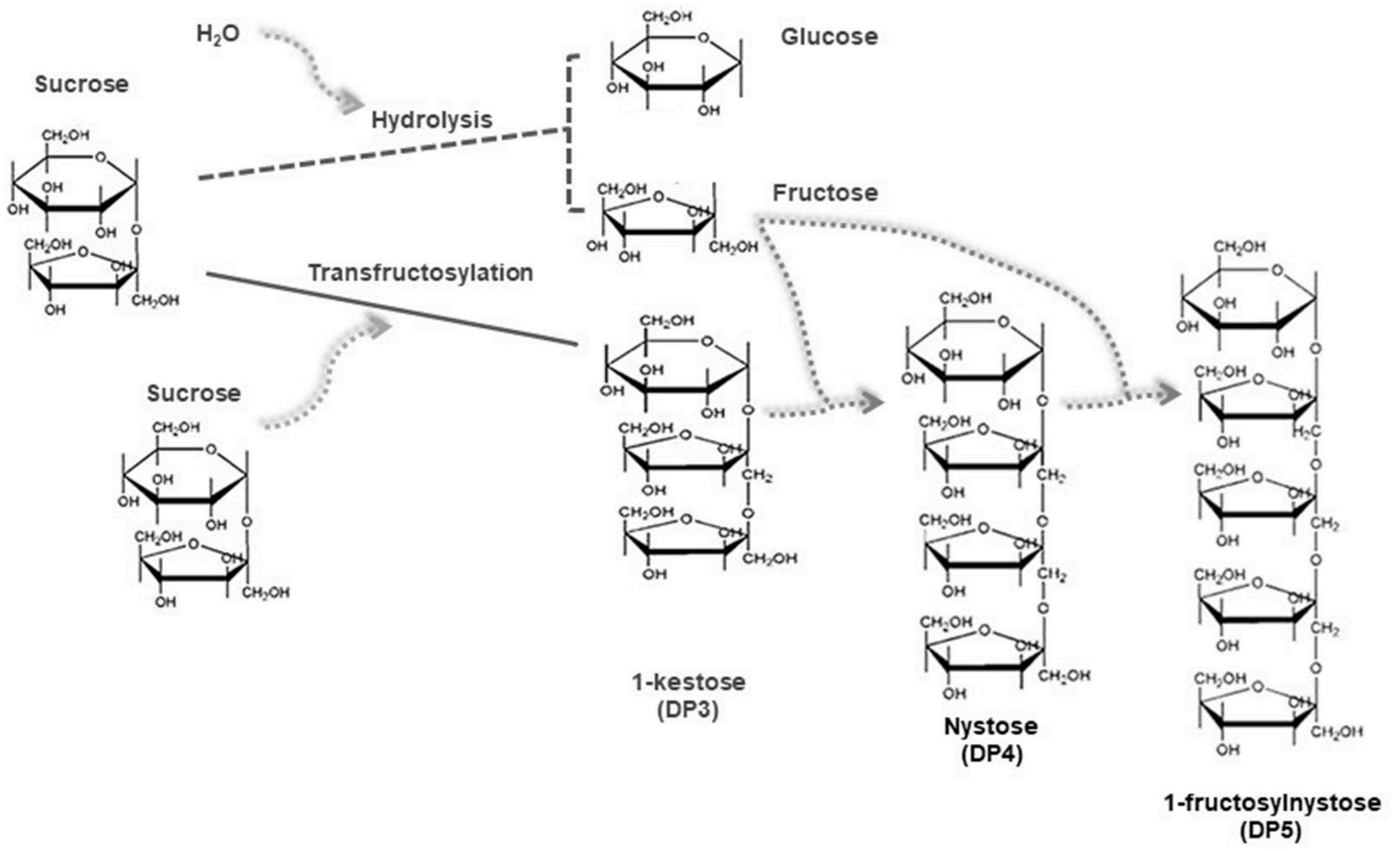
Simplified scheme for the enzymatic synthesis of FOS through transfructosylation reactions.

Transfructosylation reactions involve the cleavage of the β-2,1-glycosidic bond and the transfer of fructosyl moieties from carbohydrates acting as donors onto any acceptor other than water (17). Most fructosyltransferases have also a hydrolytic activity, so that the production of FOS is a complex process in which different reactions of synthesis and hydrolysis occur simultaneously both in parallel and in series (17), through consecutive sets of disproportionation reactions. Figure 1 gives a simplified general outline of the mechanism of the mentioned reactions. In such reactions, the FOS synthesized in the first steps act as fructosyl donors and acceptors leading simultaneously to the production of FOS with DP immediately higher (DPn+1) and lower (DPn-1) than those of the FOS acting as reagents (20). As a result, mixtures of short chain FOS (DP ranging from 2 to 6, i.e., DP3, DP4, DP5, and DP6) (4), together with glucose (secondary product), are obtained. To mathematically describe this mechanism, many authors adapted a kinetic model based on Michaelis-Menten mechanism, assuming that the series of transfructosylation reactions with sucrose, 1-kestose (DP3), and nystose (DP4) as substrates occur in chain, and also considering a competitive glucose inhibition. One of the first approaches in this sense, is the one proposed by Jung et al. (20). They described the reaction mechanism with sucrose as a substrate that can act either as donor or as acceptor, so that 1 mole of glucose and 1 mole of 1-kestose (DP3) are formed simultaneously, indicating a disproportionation reaction mechanism. This pattern was extended to explain the rest of the pathways involved in the course of the synthesis: 1-kestose (DP3) acts as a substrate and sucrose and nystose (DP4) are produced, afterwards nystose (DP4) acts as a substrate and kestose (DP3) and fructofuranosyl nystose (DP5) are formed. Applying mathematical integration of the several reaction patterns proposed, authors were able to calculate the Michaelis-Menten kinetic constant and the maximum rate of appearance of each product. Duan et al. (21), modified this mathematical model by adding the fact that glucose acts as a substrate inhibitor even for sucrose, 1-kestose (DP3) and nystose (DP4). The same kinetic approach was mathematically described by Alvarado-Huallanco and Maugeri Filho (22), using purified and non-purified fructosyltransferase from *Rhodotorula* sp. In this latter model, the authors considered that hydrolysis occurs when nystose (DP4) concentration reaches about 5% (w/v). In addition, a much lower value for the nystose hydrolytic constant was found when purified enzyme was used. In the same direction, Guio et al. (23) modified the original model (20), considering the effect of immobilized glucose isomerase, incorporated to improve FOS conversion. In addition, Detofol et al. (24) proved the accuracy of this approach both on batch and on continuous reactors. According to Vega and Zúniga-Hansen (17), this assumption just partially describes the progress of the reaction because it considers that the same substrate is acting as a donor and acceptor for the fructosyl moiety. However, the active site of fructosyltransferases contains a pocket that accommodates a single sucrose molecule in the substrate-bound structure. Therefore, they proposed a mathematical model based on a mechanism in which sucrose and FOS interact with the enzyme species applying multi-response non-linear regression. This concept was also developed by Khandekar et al. (25) who presented a five-step, ten-parameter kinetic model based on the Michaelis-Menten concept but including the step of binding sites of the enzyme, with sucrose as substrate and glucose as an inhibitor, and also the occurrence of FOS hydrolysis. These contributions regarding the mathematical models explaining the mechanisms involved in the synthesis of FOS were the most important ones reported in the last years. It is worth to mention that the more accurate the model, the better its capacity to explain technological aspects, namely the improving of reaction conditions or yield of the products.

The composition of the obtained FOS can be modulated by adjusting different parameters, namely substrate concentration, enzyme source, time, temperature and pH, all of them interacting with each other. Therefore, when searching for the optimal value of one of these parameters, the values of the other ones must also be taken into account. Table 1 summarizes several research works on this field, specifying enzyme source, reaction temperature, pH, time, substrate concentration, amount of enzyme, and the resulting FOS yield.

**Table 1:** Different enzyme sources, conditions performed, and yields for the synthesis of FOS.

**Enzyme source**	**T (^**°**^C)**	**pH**	**Sucrose (g/100 ml)**	**Enzyme amount**	**Reaction time (h)**	${\text{Y}}_{\text{FOS}\;\max}^{\text{a}}$ (gFOS/100 g sucrose)^**a**^	**Observation**	**References**
**COMMERCIAL**								
Rohapect CM (AB Enzymes GmbH)	45–60	5.5–6.5	53–72%	3.4–7.4 UT/mL^d^	3 and 5	41–64%		(15)
Viscozyme L (Blumos SA)	55	5.5	10–60%	56 FU/mL^e^	6	65–85%		(10)
25 enzyme preparations from fungal strains	45–60	4.5–6	40–80%	4.2–15 UT/mL^d^	6	59–64%	5 enzymes selected.	(16)
Viscozyme L (Novozyme)	50	5.5	60%	1,230 UT/g^d1^	2.5 (50 batchs)	40–6%	purified immobilized enzyme.	(26)
Pectinex Ultra SP-L & Rapidase TF (Novozyme)	60	5.6	63%	0.3 U/mL^f^	144	62%^h^	immobilized enzyme.	(27)
**MICROORGANISM**								
*A. japonicus* TIT-K J1	37	5.4	10, 30, 50%	0.2, 0.56, 0.96 U/mL^f^	24	65–68%		(28)
*A. japonicus*	50	5	45–70%	5.75 g cell/100 mL	4	51–59%		(29)
*A. aculeatus*	50-70	4.8–6.4	20–60%	20–100 U/mL^f^	4-24	55% DP3^h^; 43%DP4^h^		(30)
*A. niger*	55	6	10,30,60%	0.66 U/mL^f^	88	55-45%^h^		(31)
*S. cerevisiae* (invertase)	40–55	5.5	21–85%	0.5–8.0 U/mL^f^	8	10% (d.b.)^c^		(25)
*Rhodotorula* sp.	50	4.5	50–70%	5 UTF/mL	96	50–58%		(22)
*Rhodotorula* sp.	48	6	50%	0.022 U/mL^f^	48-56-72-96	44–60%	immobilized and free enzyme.	(24)
*Aureobasidium* sp. ATCC 20524	30	5.5	40%	270 U/g ^g^	20 mL/h (26 days)	1,512 g DP3	immobilized enzyme; continuous reactor.	(32)
*Cryptococcus* sp.	50	4.5	50%	1 FTA/mL	48	34%		(33)
*A. niger* IMI 303386	39	6.5	50%	0.4 U/mL^f^	72	62%^h^		(34)
*B. subtilis* natto CCT 7712	35-55	7.7	20-40%^i^	n.i.^b^	12–36	388 mg/mL^j^		(35)
Levansucrase SacB of *B. subtilis*	37	6	9%^k^	1.47 U/mL^f^	24	54%		(36)
*A. niger* AS0023	50	5.8	50%	5 × 10^6^ KU	5	62%^h^		(37)
*A. foetidus*	40–70	3–7	30%^l^	n.i.^b^	12	29–48%		(38)
*Arthrohacter* sp. K-l	40	6.5	10%	3.4 U/mL^f^	5–20	n.i.^b^	^m^	(39)
*A. niger*; *A. awamori, S. cerevisiae*	40	5	50%	6 U/g sucrose^f^	8–72	50–37%^h^		(40)
*A. niger, A. pullulans*.	50–65	4–8	70%	1:9 (w/w) cell:sucrose	8	35–38%		(41)
*A. oryzae*	55	5–6	60%	0.14 (v/v) Culture/sucrose	4–24	55%		(42)
*A. oryzae*	60	5.5	75%	275 U/g sucrose^f^	7	57%		(23)

a *Y_FOSmax_: Maximal yield of FOS*;

b *n.i. not informed*;

c *d.b.: dry basis*;

d *UT/mL: transfructosylation activity/ mL of reaction volume*;

d1 *UT/g: transfructosylation activity/g of dry support*;

e *FU/mL: fructosyltransferase units/ mL of reaction volume*;

f *U/mL: One unit of enzyme activity; the amount that produce lμmol of reduced sugar per minute/ mL of reaction volume*;

g *U/g: One unit of enzyme activity; the amount that produce lμmol of reduced sugar per minute/g of dry support*;

h *Informed yield: weight percentages of total sugar*;

i *Substrates: sucrose, sugarcane molasses and sugarcane juice*;

j *Yield informed as amount of DP4 produced*;

k *Substrates: sucrose and sucrose analogs*;

l *Substrates: maltose or sucrose*;

m *Analysis based on transfructosylated products and acceptor specificity*.

Regarding substrate concentration, in general terms, higher initial concentration of sucrose (i.e., >40%), enhances the production of shorter FOS [i.e., 1-kestose (DP3) and nystose (DP4)], with low production of glucose. On the contrary, lower concentrations of sucrose lead to the production of larger FOS [i.e., DP5 and DP6] with a higher production of glucose (10). Some authors claimed that using very high initial concentration of sucrose (85% w/v) is a technological strategy for the production of commercial syrups. This way, the final evaporation step is simplified (43). The modulation of the synthesis regarding initial substrate concentration is important to accurately obtain FOS mixtures with better prebiotic effects, taking into account that the shorter the chain length the greater the prebiotic effect (44). As stated before, the synthesis of FOS occurs through consecutive sets of disproportionation reactions in which the FOS synthesized in the first steps act as fructosyl donors and acceptors leading simultaneously to the production of FOS with DP immediately higher. Consequently, when the maximum conversion of DP_n_ is reached, it is followed by a decrease of DP_n_, leading to an increase in DP_n+1_. Taking this into account, it is crucial to know these kinetic parameters (maximum conversion and time at which is reached) to modulate the product composition, not forgetting their dependence on other reaction conditions (pH, temperature, enzyme source, enzyme concentration). In this sense, many authors have studied the effect of substrate concentration on the enzymatic synthesis of FOS, under different conditions (10, 16, 28, 29, 31, 40, 45). However, only few authors investigated the effect of more than one parameter at the same time. For example, Nemukula et al. (30) proposed a joint analysis of the effect of sucrose concentration, enzyme concentration, reaction time, temperature and pH for obtaining the maximal FOS yield, using response surface methodology. In line with such study, Vega and Zúniga-Hansen (15) studied the interaction of sucrose concentration, temperature and enzyme concentration on FOS, DP3, and volumetric yield. These approaches enabled to determine the most appropriate cost-effective condition to operate (operation temperature 50°C, pH 5.5, 6.6 TU/mL of enzyme, and 71% w/v of initial sucrose concentration), which enabled obtaining 63.8% of FOS yield (short chain FOS grams per 100 g of initial sucrose).

Concerning the enzyme source, all enzymes used for producing FOS (both by synthesis and by hydrolysis) generally belong to the glycoside hydrolases family (GH) and are either included into the GH32 or GH68 families (CAZy classification) (46). Particularly, enzymes with fructosyltransferase activity can be found in plants, yeasts and molds (GH32) and in bacteria (GH68) (47). Most commercial enzyme preparations have both fructosyltransferase and hydrolase activities; this combination gives them advantages over specific enzymes, such as low price, versatility and high stability under reaction industrial processes conditions, but the disadvantage of non-probiotic monosaccharides (i.e., glucose and fructose) being also produced as result of the enzymatic reaction (Table 1). Therefore, preparations with high transfructosylase activity are preferred for the synthesis of short chain FOS. Vega and Zúniga-Hansen (2012) (16) studied twenty-five commercial enzyme preparations from the global market (Europe, USA and South America) to obtain short chain FOS from sucrose, weighing up both transfructosylation activity and transferase/hydrolase ratio. As an example, the enzyme *Viscozyme L* from *Aspergillus aculeatus* (Novozyme, Denmark) simultaneously has high transfructosylation activity and high transferase/hydrolase ratio (16, 26, 48) (Table 1). The enzymatic transfructosylation of sucrose with bacterial or fungal fructosyltransferases (23, 33, 42, 49) or fungal β-fructofuranosidases (32, 34) have also shown promissory results. In this regard, using extracellular β-fructofuranosidases from different fungus, together with cultivation with *Picchia pastoris* increases the production of FOS DP3 (26.47%) and DP4 (57.98%) (50). Other reports describe the capacity of *Bacillus subtilis* natto CCT 7712 to produce high amounts of DP5 (nystose) from low-cost substrates, such as sucrose, sugarcane molasses, and sugarcane juice (35). Each type of enzyme was tested for FOS production under different conditions and the results and main particular observations are presented in (Table 1).

Besides the enzyme characteristics, biocatalysts can be free (10, 11, 15–17, 23, 36) or immobilized (26, 27, 51) in the reaction medium. Immobilization consists on turning the enzyme into a physically confined form in a defined region, blocking its mobility but maintaining its catalytic activity. Many authors reported a higher catalytic efficiency of enzyme membrane reactors employing free enzymes for relatively long periods (52). Although the enzymatic production of FOS using immobilized enzymes may not work optimally due to limited substrate or product mass transfer to and from the enzymes, it is a relatively new alternative, whose main advantage is offering the possibility of re-using the enzyme. This great advantage denotes the need of further research to overcome the mentioned inconvenients regarding immobilized enzymes.

Optimal pH and temperature strongly depend on the enzyme source. As it is shown in Table 1, the reaction can be performed in a widely pH range of (3–7), and the temperature can vary from (35–70°C). Nevertheless, in general terms there can be mentioned that more bounded ranges of optimal pH and temperature can be defined by gathering together more than one type of enzyme. In this respect, a large number of reports have placed the optimum pH and temperature for activity of fructosyltransferase between 4.5–6.5 and 40–60°C, respectively (30, 53, 54) (Table 1). These two parameters fundamentally affect reaction rates. In this sense, Vega et al. (16) who studied the effect of temperature reaction in a range of 45–60°C, found that the increase of reaction temperature causes an increase in the reaction rate. A similar behavior has been reported by other authors (15, 41, 55). It is important to mention that over 60°C the enzyme could present thermal damage and its activity decreases considerably (26). Regarding pH, it has strong impact in the ionization state of the constituent amino acids, thus affecting the enzyme's primary and secondary structure and consequently its activity (56). A pH of around 5.5 was reported to be optimal for fructosyltransferase production in *Penicillium purpurogenum* (57), *Aureobasidium pullulans* (58), and *Syncephalastrum racemosum* Cohn (59).

In general, the synthesis of FOS yields about 60% FOS, under the form of syrup. Most commercial FOS products are mixtures containing different amounts of FOS, products with 55–99% of purity. The presence of glucose (and residual sucrose) obtained as secondary product of reaction decreases the prebiotic effect of the mixtures, increasing their caloric and cariogenic value, and thus preventing their incorporation into health, dietetic and diabetic foods (60). To enhance the purity of FOS, mono and disaccharides can be removed. One option is the continuous removal of glucose and residual sucrose during the synthesis using enzymes and membrane reactors (61, 62). Another option is the purification process after the synthesis. There are many strategies in the research background of FOS production, but generally both activated charcoal adsorption and enzymatic methods are the most extensively used.

Purification of FOS using activated charcoal consists on the adsorption of sugars onto the activated charcoal, in a reversible process. As activated charcoal is non-polar or hydrophobic, sugars are adsorbed according to their hydrophobic character due to van der Walls forces, which is directly related to their molecular weight (the higher the molecular weight, the more CH groups and the more hydrophobic the sugar is). Hence, FOS are more strongly adsorbed than mono and disaccharides, enabling their separation (60). In practice, purification involves the filling of columns with activated charcoal (sorbent) and the re-circulation of the obtained syrups until an equilibrium between the sorbent and the moving phase is reached. After that, the non-adsorbed sugars are removed by circulating milli-Q pure water through the column. Finally, the retained oligosaccharides are recovered by elution with different ethanol gradients (40, 63–65). The products of elution are also syrups that can be concentrated and even dehydrated to obtain powders (11). The mechanisms involved in the purification of oligosaccharides using activated charcoal are determined by their molecular interactions. Packer et al. (66) deeply analyzed the efficiency of using graphitized carbon to separate oligosaccharides or their derivatives (hydrazones and alditols) released from glycoproteins from solutions containing salts (of hydroxide, acetate, phosphate), detergents (sodium dodecyl sulfate and Triton X-100), and proteins (enzymes, glycoproteins). Reagents such as hydrazine or sodium borohydride were reported to release oligosaccharides. Fractionation of neutral and acidic oligosaccharides, which are sialylated, sulfated or phosphorylated, is also possible by elution with water-acetonitrile mixtures. Although the use of such desorbents might be useful for FOS purification, the alimentary use of FOS must not be forgotten. Therefore, when FOS are to be purified, only GRAS (Generally recognized as safe) products are allowed.

Enzymatic oxidation of glucose is an alternative to purify the synthesized FOS. This method is as efficient as the former, but much easier to scale-up. The glucose can be oxidized using glucose-oxidase as biocatalyst, producing gluconic acid, which can be precipitated by the addition of Ca(OH)_2_. This calcium gluconate can be also used as source of calcium. This way, the glucose generated during the enzymatic synthesis of FOS can be transformed into other products of high added value (11). Additionally, if the synthesized FOS are treated with immobilized cells of *Zymomonas mobilis*, glucose, fructose and sucrose can be simultaneously eliminated (67). Other methods to remove mono and di saccharides from FOS syrup, are membrane technology, mainly ultra and nanofiltration (68–70), and also microbial treatment through the fermentation of glucose, fructose and sucrose to ethanol and carbon dioxide (60, 71, 72). This method involves additional process to treat fermentation products, and depending on the microorganism selected and the raw material used, additional nutrients may be necessary (67).

No matter purification process, the mixture of purified FOS still contains different concentrations of FOS with different DP. As they are usually employed in the formulation of functional foods or in infant formula, purification of each oligosaccharide is not strictly necessary. However, for mechanistic or physiological investigations, the availability of pure FOS with a given DP is necessary. The isolation is possible using preparative HPLC although it is not an easy process, especially for the production at a large scale. Indeed, pure FOS are expensive and are only available for analytical purposes.

### FOS Obtained by Hydrolysis of Inulin

In general, the presence of mono and disaccharides in the final product is one of the drawbacks of synthesis of FOS over the hydrolysis from inulin, making the yield and purity of the latter much higher. In this regard, the production of FOS using endo-inulinases yields 81%, compared to the 55% resulting from fructosyltransferases activity (73).

Plant inulin have chains of up to 60 units of fructose, which length, composition and dispersity vary with plant species, life cycle phase, time of harvest and the conditions of extraction and post-extraction. Fresh plant material is always used to extract native inulin, and precautions must be taken to inhibit the plant own inulinase activity and to prevent acid hydrolysis. Even so, the extraction of inulin is always accompanied by the extraction of FOS, sucrose, fructose and glucose in variable amounts. Inulin is soluble in water in moderate extent (about 10% at 20°C), producing a low-viscosity solution. It can form a tridimensional microcrystalline gel network at higher concentrations; this will give a fat-like mouthfeel. Inulin is about 10 times less sweet than sucrose and that sweetness is eliminated when short chain inulin molecules are removed. This process increases the gel-forming capabilities.

Commercially available inulin is currently produced by the industry from two species belonging to Compositae: Jerusalem artichoke (*Helianthus tuberosus*) and chicory *(Cichorium intybus*); however, commercial inulin from dahlia (*Dahlia pinnata*) tubers can also be found (74). Additionally, it can be extracted from the tubers of *Cynara cardunculus* (artichoke) and *Polymnia sonchifolia* (yacon) (75). Agave, garlic and shallots are also potential sources (76). Jerusalem artichoke is one of the most important raw materials for the industrial production of fructose and inulin since it is easy to cultivate, accumulates about 50–70 g/kg of its fresh weight as inulin-type fructans and the crop yield estimate is 5.4 ton/ha (77). However, both inulin contents and degree of polymerization vary extensively with time of harvest (78). This may lead to variation in the composition, something common in natural products, but a possible issue for some applications in which a very precise composition is required.

Inulin may be commercially obtained in different forms: native inulin with an average degree of polymerization (DP) of 10–12, containing short chain inulin fractions (DP 2–10) and high performance inulin (HP) with DP higher than 20. Small inulin oligomers mixture with DP <10 are often designated by oligofructose or short-chain FOS. The long-chain inulin or inulin HP is produced by physical separation techniques.

Mensink et al. (79) revised the origin, physico-chemical properties and DP of commercially available inulins. They highlight that two batches of inulin with the same average DP can have different size distributions and therefore there characteristics can be very different. Inulins with higher DP have lower solubility in water, higher melting temperatures (crystalline inulins) or higher glass transition temperatures (amorphous inulins), higher chemical stability (do not hydrolyse easily), form stronger gels and their aqueous solutions have higher viscosity.

The fructose units of inulin are linked by β-(2→ 1) D-fructosyl-fructose bonds and the chain thus formed is usually terminated with one glucose unit linked through an α-D-glucopyranosyl or α-(1→ 2) bond in the same way as in sucrose. Inulins that show this terminal glucose unit are designated by α-D-glucopyranosyl-[β-D-fructofuranosyl]_n−1_-D-fructofuranosides (FOS or GF_n_), while those that lack this glucose unit and are therefore constituted of fructose only are called fructopyranosyl-[α-D-fructofuranosyl]_n−1_-D-fructofuranosides (or inulo-oligosaccharides -IOS or FF_n_) (80).

The extraction of inulin and FOS from vegetables is carried out by grinding and solubilization in hot water, with further enzymatic treatment with sucrases (to eliminate the sucrose still present), α-amylase and maltase (for degradation of short chain carbohydrates) (81).

Enzymatic hydrolysis of inulin is the most common procedure, however other methods such as acid hydrolysis and auto-hydrolysis can also be employed for this purpose.

#### Enzymatic Hydrolysis

There are two types of hydrolytic enzymes that break down inulin, endo- and exo-inulinases. As in October 2018, BRENDA (the free comprehensive enzyme system–www.brenda-enzymes.org) has 58 endo- and 70 exo-inulinases described. These enzymes can be obtained from bacteria, fungi, yeast, and plants, although commercially available products come from the fungus *Aspergillus* spp., in particular *A. niger* (www.brenda-enzymes.org).

**Figure 2:**
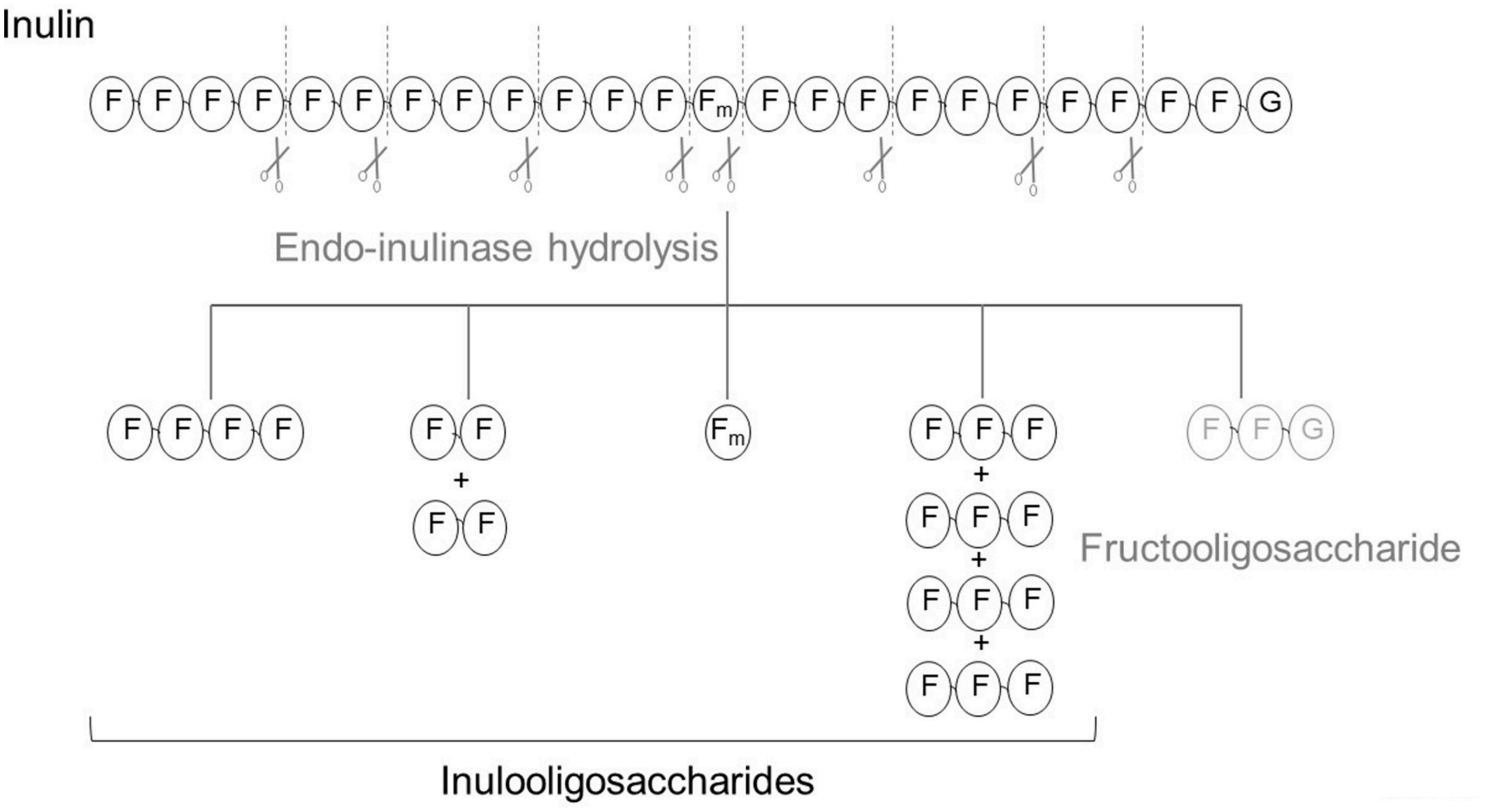
Scheme of the hydrolysis of inulin using endo-inulinase as biocatalyst. Each initial GFn yields one fructooligosaccharide and several inulo-oligosaccharides.

Endo-inulinases (E.C.3.2.1.7) are enzymes capable of cleaving linkages between fructosyl moieties/residues within the fructan chain. They have been widely used for the production of FOS, especially since the commercial inulinase form is the endolytic type (Figure 2). These enzymes can also be used to determine the overall content of inulin and FOS in plants and foodstuffs by measuring the amount of fructose, glucose, and sucrose before and after the enzymatic hydrolysis (76).

Exo-inulinases hydrolyze terminal, non-reducing 2,1-linked and 2,6-linked β-D-fructofuranose residues in inulin, levan and sucrose releasing β-D-fructose. Most exo-inulinases are capable of hydrolyzing inulin in a very effective way, producing fructose in yields as high as 90–95%, so they are used mainly for the production of ultra-high-fructose syrup.

The enzyme source can dictate the outcome of the hydrolysis: amount and type of products generated. For instance, the production of FOS using endo-inulinases from *Xanthomonas oryzae* No. 5 (82) results in FOS with DP≥5 as the major compounds, while the same enzyme from *Pseudomonas* sp. No. 65 (83) produces mainly DP2 (inulinobiose) and DP3.

In an assay with endo-inulase from *Pseudomonas* sp. No. 65, DP2 (inulobiose) and DP3 FOS were the main products (31 and 23%, respectively) when using pure inulin from chicory and, with raw chicory extract, the hydrolysate consisted of 19% DP2, 19% DP3, 14% DP4, and 19%>DP5, with fructose, glucose, and sucrose being detected in both cases. Additionally, dual systems with different endo-inulinases can also be used for the production of FOS from inulin, accordingly to the user's needs (84).

The high cost of the enzymes increases the overall cost of this process (85). This has been partially overcome through genetic engineering and molecular biology techniques, many modified enzymes with enhanced properties compared to their natural counterparts have been obtained (86–88).

Endo-inulinase genes from microbial species have been successfully cloned in another and the expressed enzyme used for the hydrolysis of inulin with noteworthy results (89). It is possible to design a system suited to the user's needs, such as high expression, intra or extracellular enzymatic production and thermoresistance. Recently, an endo-inulinase encoding gene was cloned and transfected into *Baccillus subtilis* WB800-R and the enzyme produced was used for the hydrolysis of inulin resulting in yield of around 69 g/L of FOS, mainly DP3, 4 and 5, in the crude extract, with a conversion rate of pure inulin into FOS of 75% (90).

Wang et al. (91) reported a simple and highly efficient one-step bioprocess for production of high-content FOS from inulin by yeast fermentation, using a recombinant yeast strain JZHSTSC, in which a heterologous endo-inulinase gene was expressed and the inherent invertase gene SUC2 was disrupted. This yeast simultaneously hydrolyzed inulin into FOS by secretion of endo-inulinases and removed mono-sugars by assimilation, resulting a product with high purity of FOS (~90%).

In a similar process, but in a two-step way, Han et al. (92) achieved similar results by using a recombinant *Yarrowia lipolytica* strain Enop56, in which an optimized endo-inulinase gene from *Aspergillus niger* was overexpressed. The hydrolysis in these conditions lead to the formation of FOSs with DP 3–5 as major products and to <5% of mono- and disaccharides (non-prebiotic). As before, large amounts of FFn oligosaccharides were obtained. Since both GFn and FFn oligosaccharides show identical functional and physiological properties, this is not a disadvantage (93).

Several studies of cloning and modification have been performed on fungal inulinases in order to improve efficiency, achieving yields up to 90% of oligofructose with degrees of polymerization between 3 and 6 (94).

#### Acid Hydrolysis

Glibowski and Bukowska (95) found out that heating 5% inulin solution in a strong acidic environment (pH 1–3) caused intensive hydrolysis, even mild temperatures (40°C) which somehow contradicts the notion that inulin is not digested by the human gastrointestinal tract.

It has been reported that both fructose and fructooligosaccharides can be produced from inulin by chemical hydrolysis (pH 1–2 at 80–100°C), but fructose degrades easily at low pH resulting in the formation of di-fructose anhydride, a colored byproduct with almost no sweetening capacity, and hydroxymethylfurfural, a known by-product and inhibitor for fermentative organisms.

Acid hydrolysis becomes relevant in the obtaining of FOS from agave, since the amount of fructan accumulated in the mature plants [13–17% (w/w) fresh weight] is similar to what is found in the current source of inulin, chicory [15.2–20.5% (w/w) fresh weight]. The main difference resides on the structure of the fructose polymers: while in chicory inulin fructose molecules are joined through β(2–1) linkages in linear chains, fructans present in agave, especially in *Agave tequilana*, have a relevant content of β(2–6) linkages resulting in branching fructose molecules (levan type fructans) in chains with DP 3–29. Due to their complex structures, commercial *endo*-inulinases have little hydrolytic activity over these polymers, while specific endo-levanases are difficult to obtain and fructanases, combining *endo* and exo-inulinase activities, lead fructose as the main hydrolysis product, even at low conversions. Avila-Fernadez et al. (96) used a limited acid hydrolysis by HCl and cation exchange resins for the production of FOS from agave fructans; the reaction need to be controlled to prevent hydrolysis to fructose. β-(2,6)-FOS were prepared from microbial high-molecular-mass levan by acid hydrolysis and refined by cation-exchange chromatography, resulting in oligosaccharides with a DP within 2 and 20 and the same β-(2,6) linkage type. The long-chain β-(2,6)-FOS were more resistant against acid or enzymatic hydrolysis than the short-chain β-(2,6)-FOS.

Hence, acid hydrolysis is suitable when the aim is the production of fructose syrups as an alternative to exo-inulinase hydrolysis or for bioethanol production from biomass (97).

#### Autohydrolysis

Long-term storage provides adequate conditions for the chemical breakdown of inulin and FOS. This is also the reason why older plants typically have lower inulin contents than younger ones: plants also contain enzymes that can hydrolyze inulin. The main effects are the shortening of the FOS chains and eventually the production of free sugars, that is, glucose, fructose and sucrose.

Extracted inulins may contain a large amount of sugars (mono-, di- and small oligosaccharides) (84). Typically, extraction is done by boiling the cleaned and cut or ground up tubers, or other inulin containing plant part, in water. Process conditions (pH, water–root ratio, boiling time, etc.) affect the DP of the produced inulin. Higher oligomers are more hydrolyzed than the lower oligomers, since they have a relatively high content of fructosyl end chains.

The isolation of those small oligosaccharides, which will have a glucosyl end and are thus similar to FOS obtained by synthesis, can be an interesting approach.

Cho et al. (84) found 38% of FOS (DP3 to >5) in the initial carbohydrate composition of chicory juice, together with 33% inulin and 27% mono and disaccharides. Precipitation of inulin and removal of mono and disaccharides would lead FOS as the main product.

#### Other Species Should be Considered as Direct Sources of Oligosaccharides

Benkeblia et al. (98) extracted FOS (DP3 to DP12) from onions in average amounts of 270 mg/g together with free mono and disaccharides in amounts of 450 mg/g; only the fraction of DP5-DP12 degraded with time at 20°C. Shiomi et al. (99) revised the metabolism of FOS in onions, concluding that the maximum amounts are found during dormancy, after the activity of fructosyltransferases during bulbing and before the extensive activity of exo-hydrolases that takes places during sprouting.

Yacon (*Smallanthus sonchifolius* Poepp. and Endl.) is a root crop native to the Andean region, but has also been cultivated in other regions. Yacon tubers are traditionally consumed as fresh fruit due to their crunchy texture and high juice contents, having a moderate sweet taste. Saccharides compose up to 80% of the total dry matter content of yacon tubers, with a large dependence on cultivar. These saccharides consist of fructose, glucose, sucrose and FOS, which are usually as their dominant group of saccharides (100).

Campos et al. (101) studied 35 different yacon accessions and found that the content of reducing sugars (RS), sucrose (S) and FOS based on dry matter vary wildly depending on accession. The highest FOS contents found was 65.0 g FOS/100 g DM. The content of RS in yacon accessions was inversely correlated to the FOS content.

Sumiyanto et al. (102) analyzed the fructans content in tuberous roots of yacon and found values between 70 and 80% of the dry weight during the harvest period of October-December and very little variation in the amount of other solids over this period of time.

The fructooligosaccharides in yacon represent mainly oligosaccharides from DP3 to DP10 with terminal sucrose (inulin-type fructooligosaccharides) (103). Regarding other nutrients of yacon, many studies reported that it contains low protein, lipid and ash content, thus making this tuber a potential source of FOS.

The large variations in mono and disaccharide content may be due to the accession, edaphoclimatic conditions during growth of yacon, and particularly the post-harvest procedures. A common postharvest strategy consisting on exposing the tubers to direct sunlight in order to increase their sweetness will cause the breakdown of FOS to FOS with lower DP and/or free fructose and glucose. Processes such as drying will also modify the profile of carbohydrate content of yacon tubers (104).

A derivative of yacon that is industrially available is yacon syrup, produced by juicing the fresh roots, filtering and concentrating by evaporation of water (105). Since the water contents is diminished to about 20%, the syrup can be stored for several months without significant reduction of FOS content or significant depolymerization (106).

The enzymatic hydrolysis of inulin generally produces chains longer than DP5, with a lower prebiotic activity than those produced synthetically (DP3-5). Depending on the application of the generated FOS, these points should be considered (90). There is the possibility of making use of the action of exo-inulinases (E.C. 3.2.1.80), which remove frutosyl residues from the non-reducing end of the inulin molecule, thus shortening the chain, but these also hydrolyze sucrose and raffinose, thus produces a mixture containing high amounts of free glucose and fructose, since this enzyme is able to hydrolyze the glycosidic bond α-(1,2), which connects glucose to the main inulin chain, so additional steps of purification are also needed.

The major drawback of inulin as a source of FOS is the fact that it is not a single structure. The chemical structure of fructans vary widely depending on the species. For example, as mentioned before, inulin from some plants has a 2,1-linked -D-fructosyl back bone with 2,6-linked -D-fructosyl side chains in variable percentage, as in garlic and *Agave tequilana* (96), while other have only linear chains. Degree of polymerization differences are another issue: inulin from chicory (*Cichorium intybus*) has a much lower DP (about 20) than inulin obtained from globe thistle (*Echinops ritro*) with mean DP 30 or global artichokes (*Cynara scolymus*) with mean DP65.

The species mentioned in this section also contain their own inulinases, which is the major drawback for inulin recovery. Leroy et al. found that throughout the period of artichoke storage, a decrease in inulin content and mean DP occurs, owing to its *in natura* depolymerization (107). *A. tequilana* was investigated as potential inulin source, the youngest plants exhibited the highest levels of free monosaccharides and low molecular weight fructans with potential application as prebiotics, while the DP reached a maximum of 3–30 in 4-year-old plants and then decreased to 4–24 in the oldest (>6 years) ones (108).

Another important issue is the need to extract and purify inulin from the natural matrix usually requires juices extraction and a succession of freezing, thawing and (ultra)centrifugation in order to remove low DP and other contaminants. Filtrates are deionized by passing through strong anionic and cationic resin exchangers, before a final step of freezing/thawing/centrifugation. As a result, very pure inulin (>98% purity) can be obtained. Depending on the application, this process, albeit tedious and costly, can be very effective specially to obtain FFn oligosaccharides from long chain inulins, since each resulting molecule contains a terminal glucose (GFn) for several FFn oligomers after high purity endo-inulinase hydrolysis. The general mode of endo-inulinase action is that the hydrolytic activity for inulin increases with the degree of polymerization of fructosyl residues (73). For global artichoke, F_3_ and F_4_ were the main fructose polymers (80).

However, Cho et al. (82) carried out IOS production from chicory root juice, using endoinulinase from *Xanthomonas oryzae* No. 5, and compared with FOS from pure inulin. From their results, hydrolysis with endo-inulinase over the extract does not affect ≤DP4, mainly converting inulin in DP5 and >DP5. So, if the aim is the use of FOS, the initial purification on inulin is not necessary and the removal of mono and disaccharides could be left as final step.

Physical techniques, such as ultrasound, have also been reported as methods for the production of low molecular weight FOS fragments from Jerusalem artichoke inulin extractions (109). Furthermore, ultrasound extraction of *Flammulina velutipes* polysaccharides has also been reported as a method for production of FOS (110).

## GOS

GOS are composed by a variable number of galactose units, within 2 and 10. Similarly to FOS, GOS can be obtained either by synthesis or by extraction and hydrolysis. The type of linkage between units varies according to their origin and obtaining process. Plant based GOS are α-GOS whereas GOS prepared from lactose are β-GOS.

α-GOS are important components of seeds, namely pulses, and show a terminal sucrose unit and the linkage between monosaccharide moieties can be [Gal-α(1→ 6)-Gal], [Gal-α(1→ 4)-Gal], [Gal- α(1→ 3)-Gal] and [Gal-α(1→ 6)-Glu-β(2→ 1)-Fru]. This is called the raffinose family (RFO). Another relevant α-GOS is melibiose, a reducing disaccharide with a linkage (Gal-α(1→ 6)-Glc) (isomer of lactose)

β-GOS, also known as oligogalactosyllactose, oligogalactose, oligolactose, transgalactosylated oligosaccbaride, and transgalacto-oligosaccbaride, show a terminal glucose unit and the galactose units are linked mostly by β(1→ 4) and β(1→ 6) bonds (111–113).

Although tri- to hexa-saccharides, with 2 to 5 galactose units (DP3-6), tend to be the main components of GOS-containing products, disaccharides (DP2) consisting of galactose and glucose with β-glycoside bonds such as [Gal-β(1→ 6)-Glc], [Gal-β(1→ 6)-Gal], [Gal-β(1→ 4)-Gal] or [Gal-β(1→ 3)-Gal] which are different from lactose, [Gal-β(1→ 4)-Glc], are also present and defined as GOS since they have physiological characteristics are similar to longer chains.

The prebiotic effect of α- and β-GOS is mainly associated to tri and tetrasaccharides (DP3 and DP4, respectively) (114–116).

### GOS Obtained by Enzymatic Synthesis

GOS can be commercially synthesized from lactose through transgalactosylation reactions, using β-galactosidases (EC 3.2.1.23) as biocatalysts (117). The main companies leading GOS market are Yakult Honsha Co Ltd. in Japan with Oligomate 55 syrup and Oligomate 55P powder (both with 55% dry matter of oligosaccharides), and TOS-100, a purified powder containing 99% oligosaccharides (118). Also, Friesland Foods Domo in The Netherlands commercializes TOS-syrup (75% w/v content of GOS) and Vivinal GOS syrup (with 75% w/v of solids which 59% are GOS (4).

α-GOS can also be produced by transgalactosylation reactions of α-galactosidase (α-Gal) or by conversion of raffinose family oligosaccharides by levansucrase. However, there is very little data on transgalactosylation reactions of α-Gal (119, 120), and therefore, all the discussion will be based on the better known β-GOS production.

**Figure 3:**
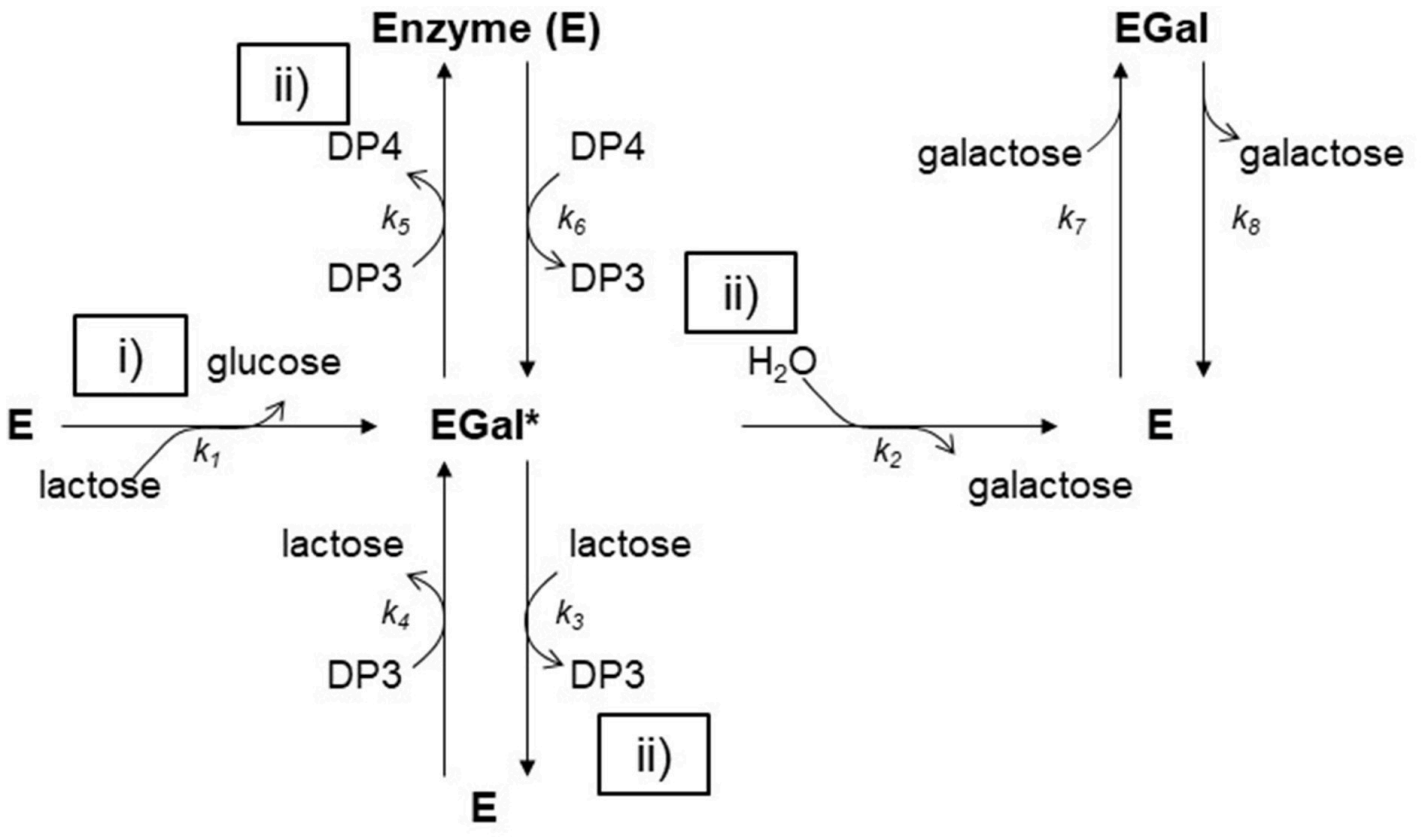
Reaction mechanism for the hydrolysis and transglycosylation of lactose by β-galactosidase. (i) The lactose molecule on the active site of the enzyme forms an acyl-enzyme complex with liberation of glucose; (ii) The enzyme-galactose complex, can react with carbohydrate molecules.

β-galactosidases from fungi of the genus *Aspergillus* and yeasts of the genus *Kluyveromyces, Rhodotorula, Bullera singularis* and *Sterigmatomyces*, as well as bacteria of the genus *Lactobacillus* or *Bacillus* are generally used as biocatalysts for the industrial synthesis of GOS (121, 122) both for food and pharmaceutical applications (123). These enzymes are widely known for their glycoside hydrolase activity, leading to the cleavage of β-galactosides into monosaccharides. However, in certain conditions, they can be used as biocatalysts for the synthesis of GOS. Indeed, β-galactosidases identify different types of glucose-glucose bonds [i.e., β(1→ 2), β(1→ 3), β(1→ 4)], as well as β(1→ 6) and β(1→ 3) glucose-galactose bonds, and catalyze the transfer of a galactose moiety from a β-galactoside to an acceptor containing a hydroxyl group. The accepted mechanism for the enzymatic catalysis involves two steps (Figure 3):

i- The formation of an enzyme–galactosyl complex, with simultaneous liberation of glucose;
ii- The transfer of the enzyme–galactosyl complex to a nucleophilic acceptor containing a hydroxyl group. If the nucleophilic acceptor is water, galactose is obtained as a product (hydrolysis reaction) (Figure 3) (124). If the nucleophilic acceptor is another sugar, di, tri or higher DP GOS are produced (Figure 3). The mechanism has been mathematically described by many kinetic models. Boon et al. (125) reported that the best approach for describing GOS synthesis by β-galactosidases is a kinetic model that considers lactose hydrolysis and oligosaccharide synthesis, so there must be taken into account that water or lactose can attack the galactosyl-enzyme complex, and also it must be included glucose inhibition. From a mathematical viewpoint, the problem can be raised by integrated rate equations and fitted by non-linear regression at different concentrations of substrate (126) so each parameter can be estimated separately and independent of the initial lactose concentration (127).

High concentrations of lactose compete with water for the transfer of galactosyl moieties (ii). Therefore, under these conditions β-galactosidases catalyze the formation of GOS (128). On the contrary, lower concentrations of lactose promote lactose hydrolysis rates to glucose and galactose (112). To stimulate the synthesis of GOS, two main approaches are used: the equilibrium approach and the kinetic approach. Both approaches tend to favor transgalactosylation over hydrolysis, the former through high substrate concentration (less water available in the medium) and the adequate enzyme/substrate ratio, depending on the enzyme source (122) and the later through enhancing the kinetic variables that promotes the most favorable rate of product formation (129).

β-galactosidases are the most frequent catalysts used in the synthesis of GOS, although their main application is the hydrolysis of lactose to generate products suitable for lactose allergic people (129). Different species possess different specificities for building glycosidic linkages and therefore produce different GOS mixtures. For example, the β-galactosidase from *K. lactis* produces predominantly β-(1→ 6)-linked GOS, the β-galactosidase from *Aspergillus oryzae* produces mainly β-(1→ 3) and β-(1→ 6) linkages (130), *Bacillus circulans* β-galactosidase forms mainly β-(1→ 4)-linked GOS (131), whereas β-galactosidases from *Lactobacillus* spp. preferably form β-(1→ 6) and β-(1→ 3) linkages in transgalactosylation mode (132, 133). Another important factor regarding enzyme source is the maximum GOS yield and the lactose conversion, that is, the percentage of initial lactose that is consumed during the synthesis. This latter is a very important factor because it has very important nutritional and technological consequences (both the hydrolysis -monosaccharides- and the synthesis products -GOS- are much more soluble than lactose, thus it is possible to go from a suspension to a syrup during the enzymatic reaction). The decrease in the lactose concentration is desirable in people with lactose intolerance. Just to mention some examples, β-galactosidase from *Aspergillus oryzae*, yields 28% of GOS with a lactose conversion of 58% (112), β-galactosidase from *Bacillus circulans* yields 54% GOS (134), and β-galactosidase from the thermophilic archaeon *Thermus caldophilus*, 75% GOS with 50% of lactose conversion (118).

Besides the type of enzyme, generally, the reaction conditions (i.e., initial substrate concentration, temperature, pH or presence of inhibitors or activators of the enzyme) affect the enzyme activity (135, 136). For this reason, all these parameters strongly determine the yield and composition of the GOS obtained, as well as the concentration of mono and disaccharides present in the products of reaction. Table 2 presents a detailed list with enzymes from different origins used for GOS synthesis, together with the respective reaction conditions and yields. As each type of enzyme has different optimal conditions (lactose concentration, pH, time, temperatures), they have to be deeply investigated to achieve the best performance (lactose conversion, yield of GOS) to obtain the desired composition of GOS.

**Table 2:** Different enzyme sources, conditions performed and yields for the synthesis of GOS.

**Enzyme source**	**T(^**°**^C)**	**pH**	**lactose (g/100 mL)**	**Enzyme amount**	**Reaction time (h)**	**Y_**GOS max**_ (gGOS/100 g lactose)^a^**	**Observations**	**References**
**COMMERCIAL**								
Maxilact 2000 (*K.lactis*, DSM); Lactozym 2000L (*K. fragilis*, Novozyme)]; Ha-Lactase (*A. oryzae*, Chr. Hansen)	40	6.5	10, 20, 30%	40 GAU/g^b^	1	14%^h^		(129)
Biolacta FN5 (*B. circulans*, Vitachem)	4–60	6.6	10 and 5% (skim milk)	0.1 pNPG/g^c^	0–32	54%		(134)
Biolactasa-NTL CONC X2 (*B. circulans*, Biocon)	60	6	50%	40 IU/g^d^	5	39%	Free and immobilized enzyme.	(137)
Lactase *(A. Oryzae*, Enzeco Fungal)	40–55	4.5	40–60%	5–300 IU_T_/g^e^	10	17–30%		(138)
*A. oryzae* (Merck), Lactozyme 2600 L (*K. lactis*, Novozymes), strain K12 (*E. coli*, Worthington)	35	4.5–7	30%^i^	50 U/g^b^	12	25%	3 enzymes compared	(139)
*E. coli* (Sigma-Aldrich)	10–60	5.5–8	2.5–15%	4.5, 9,14 U/mL^f^	24	49%	^j^	(140)
*A. oryzae* Genencor International	40	4.5–6	5–50%	4.5 g of support	37 mL/h (2 days)	25%^h^	Immobilized enzyme, continuous reactor.	(130)
*A. oryzae* (Enzeco Fungal Lactase)	50	4.5	20%	388, 250,100 IU_T_/g^e^	2.5	33–47%	Immobilized enzyme	(141)
*A. oryzae and K. lactis* (Sigma), *Bacillus sp*. (Taiwan Fructose Co.)	30–50	5	34%	4.5–10 U/g^g^	18	n.i^b^		(142)
**MICROORGANISM**								
*Sulfolobus solfataricus*	70–90	5–7	30–60%	1.2–4.8 U/mL^f^	48–60	50–53%		(143)
*S. solfataricus* and *Pyrococcus. furious*	70	5.5	4.5–17%^i^	1–2 U/mL^f^	200	50%	Continuous and batch reactor.	(144)
Bifidobacteria (BbgIV)	45–65	6.5	43%	10 U/g ^g^	24	49–53%^h^	Free and immobilized enzyme.	(145)
*Lactobacillus delbrueckii* subsp. *bulgaricus*	30-50	6.5	20%	1.5U/mL^f^	5, 8, 12	48.2–49.5%^h^		(133)
*Rhodotorula minuta* IF0879	60	5	25%	0.24 U/mL^f^	50	39%^h^		(146)
*P. acidipropionici* and Lactozym®Pure 6500L (Novozymes)	45	6.5	30%^k^	1.3 U/mL^f^	24	24%^h^		(147)
*S.thermophilus* DSM2 0259	37, 50	6.5	65%^i^	2.7 U/mL^f^	9	50%^h^		(148)
*A. oryzae* and *C. laurentii A. oryzae* and *K. lactis*	55 45	4.56.4	20%	1 IU/g ^d^50 IU/g ^d^	3-96	33-34%25%	Combination of enzymes	(149)
*L. reuteri*	25–37	6, 6.5	13.5, 30, 60%	195 U/g	70	38%		(132)

a *Y_GOSmax_: Maximal yield of GOS*;

b *GAU/g: the amount of enzyme which releases 1 μmol of O-nitrophenol per minute/ g lactose*;

c *pNPG/g unit of para-nitrophenol galactoside/ g lactose*;

d *IU/g: theamount of enzyme producing 1 μmol of O-nitrophenol per minute/g lactose*;

e *IU_T_/g: the amount of enzyme that catalyzes the transglycosylation of 1 μmol of galactose per minute*;

f *U/mL: theamount of enzyme producing 1 μmol of O-nitrophenol per minute/mL substrate solution*;

g *U/g: amount of β-galactosidase needed to liberates 1 lmol glucose per min/g lactose*;

h *Informed yield: weight percentages of total sugar*;

i *Substrate: whey permeate*;

j *Glucose and galactose (10 or 50 g/L) were added to evaluate inhibition effect*;

k *Substrate: lactose or lactulose*.

Similar to the synthesis of FOS, the initial concentration of lactose determines the chemical composition of the synthesized GOS, the more concentrated the substrate, the larger the synthesized GOS (129, 142). Some authors (150) claimed that the initial lactose concentration is directly related with the enzyme activity explaining that higher concentrations favor an increase in GOS yield (121, 127, 138). However, Adamczak et al. (129) investigated the effect of different lactose concentrations and commercial enzymes (Table 2), concluding that the lowest lactose concentration used (10%) was the one resulting in the greatest GOS yield 13.7% when Ha-Lactase from *Aspergillus oryzae* was employed as a biocatalyst. Besides, although the solubility of lactose in water is rather low (220 g/L at 25°C (151), this is not a limitation for the synthesis of GOS. Even when suspensions of lactose with constant shaking can be used as a substrate, the employ of thermostable enzymes enables the synthesis at higher temperatures, which also increases the solubility of lactose. Also, Gosling et al. (134) used a commercial enzyme preparation and 5 and 10% w/v lactose as a substrate, achieving a yield of 50% of GOS regarding initial lactose content. In this sense, Petzelbauer et al. (144) achieved high conversions of lactose into GOS by using a thermostable enzyme that allows to operate at 70°C, thus allowing a continuous hydrolysis of lactose (Table 2). Moreover, it was reported that when GOS synthesis was carried out with saturated lactose solutions, the specific enzyme productivity increased while maximum yield slightly decreased with temperature (138). When partially dissolved lactose was employed, an increase in temperature produced an increase in both yield and specific productivity (138). In addition, the continuous removal of the synthesized GOS drives the reaction over time to consume different concentrations of lactose (152). At this point, it should be pointed out that in spite of the several attempts to counterbalance the low solubility of lactose, it must not be forgotten that lactose is a very cost-effective substrate and its price is not a limiting factor for the synthesis of GOS. Only when the lactose used as a substrate takes part of a more complex matrix, such as when using milk or whey permeate, the effect of higher temperatures should be especially considered. In such cases, thermostable enzymes are a good strategy to enhance GOS yields, but the increase in reaction temperature during synthesis must be controlled, as Maillard reaction can occur due to the presence of amino side-chains of proteins and sugars (150).

The reaction temperature is directly related with the lactose concentration (lactose solubility, as mentioned before) and the stability of β-galactosidases (stability of enzymes). During the last decades increasingly interest have raised to find thermostable and thermoactive versions of β-galactosidases (153–157). One of the main enzymes used for the synthesis of commercial GOS is BgaD, obtained from *Bacillus circulans*, and used for the synthesis of GOS commercialized as Vivinal (Orafti), BiOligo® (Ingredion) Purimune™ and Yakult Oligomate 55®. The enzyme is stable up to 65°C (optimal temperature *ca*. 60°C), thus enabling high lactose concentrations (Table 2). Other thermostable β-galactosidases (recombinant) were studied even at temperatures above 80°C (143, 158). These enzymes showed an increase in reaction yields given that higher temperatures favor higher rates, high lactose solubility, and favorable equilibrium for transgalactosylation reactions (144, 159).

Regarding pH, several studies claimed that the optimal pH for GOS production is in a range of 6–7 (143, 160–163). However, a more certain pH value must be adjusted considering the enzyme source (150) (Table 2). In particular, commercial β-galactosidase from *Aspergillus oryzae* is more efficient in acid than in neutral solutions. Nevertheless, Rodrigues Mano et al. (139) confirmed that transgalactosylation activity for this enzyme have a stronger dependence on lactose concentration than on the pH of the solution.

Experimental research outlined that galactose and/or glucose commonly act as inhibitors for many β-galactosidases. Although galactose is recognized to have a greater inhibitory effect than glucose because it directly competes with lactose to form the galactosyl-enzyme complex (150, 153, 164, 165), this issue is quite controversial and strongly depends on the enzymes and reaction conditions. There are reports showing that for some enzymes neither glucose nor galactose are inhibitors (122), some enzymes have only galactose as inhibitor (165), and some others are inhibited by both sugars (158). As galactose is a competitive inhibitor of most of the β-galactosidases (especially in the hydrolysis of lactose), high concentrations of lactose can counterbalance this inhibitory effect (113). On the contrary, galactose is used to enlarge the chains of GOS during the transgalactosylation reaction (113). Glucose was claimed to have a greater inhibitor effect in some cases (125) and to have similar inhibitory effect (166) respect to galactose. In this regard, glucose is an inhibitor of β-galactosidases from *Lactobacillus reuteri* (132), *Sulfobacterium solfataricus* (155), *Thermus* sp. (167), *Kluyveromyces lactis* (168), *Thermus* sp. (169), and *Caldicellulosiruptor saccharollyticus* (158). As the inhibitory effect of glucose mainly occurs during GOS production (113). Therefore, the desirable enzymes are those with low inhibition of lactose hydrolysis by glucose. The inhibitory or activator effects of glucose and galactose are also dependent on the enzyme source and on the concentration of reagents and products (170). Hence, β-galactosidase from *Kluyveromyces fragilis* was reported to be affected by both combined and individual effects of lactose, glucose and galactose. Glucose is an activator at low concentrations of lactose and galactose and an inhibitor at higher concentrations of these sugars. In turn, galactose becomes an activator of the enzyme at high concentrations of glucose and low concentrations of lactose.

The enzyme is one of the major cost factors for the synthesis of commercial GOS. Therefore, immobilization of β-galactosidases deserved great attention in the last decade, as a way to improve their stability, enable their reutilization and facilitate their removal from the reaction medium. All these advantages enhance the yield of GOS in relation to the enzyme concentration (higher g GOS/ IU of enzyme). Immobilization technique requires a carrier that interacts with the enzyme through physical adsorption, entrapment or covalent binding (171, 172). Different parameters define the efficiency of the support, namely mechanical resistance, enzyme interaction, particle size, specific surface area, among others. Regarding mechanical properties of the support, they rather depend on the final configuration of the reactor than on the application. For example, for a fixed-bed reactor, rigidity is a desired characteristic for the support to bear high pressures, thus, silica-based materials, carbon materials, porous glass, and other mineral materials are good choices in this case (173). On the other hand, if the process is carried out in a stirred-tank reactor, flexible materials (i.e., agarose beads, cellulose beads, Lentikats -polyvinyl alcohol polymers shaped like a lens-) are more adequate (174). With respect to enzyme interaction, physical adsorption on different scaffolds (i.e., cellulose, starch, charcoal carbon, diatomeaceus earth, Shephadex, cotton cloth, chitosan) has the advantage of being cost-effective with little influence on the enzyme conformation (171). Although the weakness of the binding forces represents a disadvantage of these methods, a treatment with glutaraldehyde can stabilize the enzyme adsorption. In what concerns entrapment methods, enzymes are enclosed in polymeric matrices (i.e., alginate beads, carrageenins, polyacrylamide) or in membranes (i.e., nylon, cellulose, polyacrylamide). These methods are simple and mechanically resistant but enzyme desorption is more difficult compared to the physical adsorption, and requires cross-linking (172). Finally, covalent binding scaffolds establish covalent bonds with the functional groups of the enzyme (amino, carboxyl, hydroxyl, and sulfydryl groups), taking care of protecting the active site. They include eggshell, nylon, zeolite, gelatin, and Sephabeads-epoxy for thermo-stable enzymes. Particle size is another factor to consider depending on the operative characteristics of the synthesis. In general, large particles may be retained more easily than small ones, but they may produce preferential ways in column reactors or present diffusional problems given that long pores may decrease the rate of enzyme adsorption. At last, pore size and specific surface area of a porous particulate support are related parameters: in general, the larger the pores, the smaller the specific area. There must be reached a compromise solution considering loading capacity and size of protein/substrates (175).

For β-galactosidases immobilization several scaffolds were analyzed depending on the enzyme source, both in batch or in continuous operations, and reactions were carried out within 37 and 55°C and pH within 3 and 6.5. Enzymes from *A. orizae* were immobilized in covalently bound cotton cloth (130), in activated chitosan (83, 141, 176, 177), in the form of self-supported cross-linked aggregates (178), in amino-epoxy sepabeads (141), in glyoxyl agarose (137, 141), in magnetic polysiloxane–polyvinyl alcohol beads (127), in magnetic particles coated with polyaniline (179) in magnetite nanoparticles (176). In turn, β-galactosidases from *Bacillus circulans* were immobilized in epoxy-EupergitC (180, 181), in microporous polyvinylidene fluoride or polyvinylidene difluoride (PDVF) membrane (182), or in activated agarose (137). Finally, enzymes from bifidobacteria were immobilized in DEAE-cellulose (145), Q-Sepharose (183), amino-ethyl agarose (184). Enzymes from *Kluyveromyces lactis* were immobilized in glutaraldehyde activated chitosan (185) or in the form of whole permeabilized cells containing the enzyme (186, 187) and enzymes from lactobacilli, in microcrystalline cellulose (188), in PVC silica sheets, active carbon, porous glass beads (189). Among all these strategies, the immobilization in activated agarose (137, 141), in activated chitosan (176, 177), in magnetic polysiloxane–polyvinyl alcohol beads (127), in the form of self-supported cross-linked aggregates (178), and in the form of whole permeabilized cells containing β-galactosidase (186) appear as the most promising ones in terms of maximum yield of GOS and highest productivity (gGOS per liter per hour) (113).

Beyond all these reaction parameters and immobilizing strategies that can be modulated to enhance enzyme activity, the yield of GOS resulting from the enzymatic reactions is in general, relatively low. These can be easy deduced by comparing Table 1 with Table 2. The maximum GOS yield regarding the initial lactose concentration rounds 50% (Table 2) while that of FOS regarding initial sucrose concentration often overcomes 60% (Table 1). Moreover, their composition, both in type of linkage and molecular size distribution strongly depends on the enzyme used (190). Glucose, galactose and lactose that did not react are the main secondary products of the enzymatic reactions. Likewise in the synthesis of FOS, they shall be removed.

To this aim, similar chromatographic methods such as size exclusion chromatography (191–193) and charcoal-celite chromatography (150, 194) have been proposed.

Selective fermentation is another strategy to remove monosaccharides (135, 142, 194, 195). It basically consists on an anaerobic glycolysis by yeasts, in which the monosaccharides are converted into ethanol and CO_2_ (72). This method has the advantage that can be performed directly during the synthesis, and the disadvantage that removal of yeast cells and ethanol are necessary to obtain the purified GOS (135).

Another technology available for GOS purification is ultrafiltration (122), a process where fluid containing enzyme and product flow at a high rate across a membrane surface at a certain fluid pressure. Commonly, membrane pore-size is designed to retain the enzyme while smaller molecules (GOS) are permeated (13). Given that ultrafiltration usually does not ensure the complete elimination of monosaccharides (low molecular weight), nanofiltration appears to be a potential industrially scalable method for purification and concentration of oligosaccharide mixtures (196–199).

Additionally, *in situ* adsorption or precipitation of the undesired sugars (194, 200) are other alternatives for the removal of glucose and lactose. More recently, using immobilized enzymes enabled the simultaneous synthesis of GOS and elimination of mono and disaccharides (201). To this aim, β-galactosidase from *Aspergillus oryzae* was immobilized in glyoxyl-agarose of different particle sizes (fine and macro). At higher lactose concentrations, the hydrolytic potential of the enzyme was of 16 and 30%, and the ratio of transgalactosylation to total reaction, 70–84%.

### Obtaining of GOS by Hydrolysis From Vegetal Matrices

**Figure 4:**
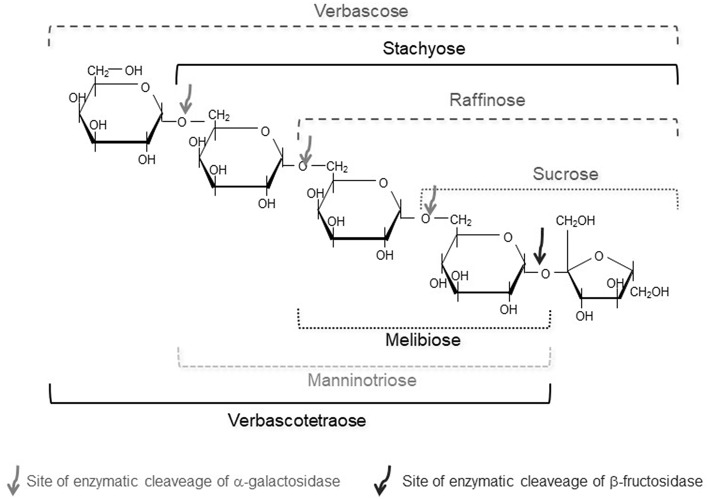
Structures of α-GOS with (Raffinose family, RFO) and without a terminal fructose.

Plant based GOS, with α-galactosidic linkages instead of β- ones, are vastly distributed and ubiquitous in the plant kingdom (202). Raffinose, a trisaccharide (Gal-Glc-Fru) is the smallest RFO. Further elongation with Gal residues leads to the DP4 stachyose (Gal-Gal-Glc-Fru), verbascose (DP5), ajugose (DP6), etc. (Figure 4) (203). Relevant amounts of α-GOS occur especially in generative parts of plants, such as seeds and fruits; GOS have diverse functions such as physiological protection, germination inhibition under low water availability conditions, and play a role in cold acclimation of many plants (204).

Sugars belonging to the raffinose family have been implicated as protective agents in the cellular dehydration tolerance in plant seeds. Experiments on liposome preservation have demonstrated that the effect of degree of polymerization since RFO were progressively better to stabilize liposomes against leakage of aqueous content and against membrane fusion after rehydration, due to the higher glass transition temperature of the longer chain oligosaccharides (205).

α-GOS can be obtained by extraction from plants, mainly from legume seeds (pulses), such as soybean, lupin, lentil, chickpea, pea and cowpea. α-GOS from soybean are the only legume oligosaccharides in the market and the main producer is Japan. More recently, a French company, Olygose, has developed a type of GOS called Alpha-GOS®. Previously, this compound was a by-product of pea protein production. After research was conducted on the effectiveness of GOS as a prebiotic, Olygose began to produce Alpha-GOS® intentionally, from peas sourced from local farmers in France.

Extractable amounts vary from 1 to 10%, depending on species and cultivar (206, 207). Espinosa-Martos found that GOS content of soybean seeds vary with the degree of maturity. Immature seeds contain less amount of GOS than fully matured ones, but no influence of biological or intensive agricultural practices in GOS content were reported (208).

Unlike FOS, there is no inulin equivalent (no long polymer) from which GOS could be obtained by hydrolysis. DP3 and DP4 are the most abundant GOS but chains of DP7 have been extracted from chickpeas. Usually sucrose is extracted along GOS which some authors claim can be purified by ethanol precipitation (209). However, others found no evidence that sucrose and soy galacto-oligosaccharides could have a differential behavior, both having a similar distribution between the two eluents: water and 70% ethanol. Kim et al. (210) optimized the conditions for oligosaccharide extraction and evaluated an ultrafiltration system for the purification of galacto-oligosaccharides from defatted soybean meal. Their main conclusion was that their system was more efficiency in the removal of protein than in the concentration of oligosaccharides, and no different distribution of GOS and sucrose is observed.

Both extraction and purification procedures must be optimized for each matrix, based on its composition. Considering that seeds are usually rich in lipids, a deffatening step must be performed prior to sugar extraction. Seeds are also high protein parts of the plant and soluble proteins and peptides are normal heavy contaminants on a first extraction. Soluble fiber, such as pectins, are also present on the aqueous extracts (211).

The viability of industrial production of GOS by extraction from natural sources depends strongly on the demands of the application, concerning purity. In order to achieve high purity, a complex set of procedures must be implemented, each step leading to loss of yield.

Like β-GOS, α-GOS are not hydrolyzed in the upper part of the human gastrointestinal tract, due to the absence of the enzyme α-galactosidase. In the colon, they are fermented together with soluble dietary fibers by the colon microbiota, generating significant amounts of short-chain fatty acids (212). These fermentation substrates stimulate the growth of lactobacilli and bifidobacteria and the decrease of enterobacteria in the intestinal microflora. This prebiotic action is beneficial for the host's well-being and health (213, 214). However, fermentation also produces gases (carbon dioxide, hydrogen and methane) that generate bloating and flatulence. Indeed, GOS of RFO are considered an important factor in the development of flatulence caused by consuming legumes (215). On the contrary, melibiose did not promote gas formation, thus suggesting that the fructose moiety present in raffinose was responsible for the gas production (Figure 4) (216).

Recently, research has been devoted to so-called “alternative” RFOs in plants. These are novel plant GOS that did not get much attention in the past. The stachyose derivative manninotriose (Gal-α(1→ 6)-Gal-α(1→ 6)-Glc) (Figure 4) was found for the first time as main carbohydrate in a garden weed *Lamium purpureum* known as deadnettle (217).

This non-fructosylated raffinose family of α-GOS includes melibiose, manninotriose and verbascotetraose and has been found naturally in foodstuffs (218–220). *In natura*, they may be the result of the activity of plant acid invertases (β-fructosidases), which are able to split sucrose into fructose and glucose by hydrolysis of the 2→ 1 glycosidic bond. This appears to be the base of the recent commercial product of Olygose, mentioned before, that starts with RFO from peas and uses invertases to remove the terminal fructosyl unit from the α-GOS chain.

The European Food Safety Authority (EFSA) analyzed the claim that this group of α-GOS, like RFO and β-GOS, is resistant to hydrolysis and absorption in the small intestine and decided in favor (221); at the same time, they do not have the same gas production negative effects.

## Other Substrates for the Synthesis of FOS and GOS

Nowadays, Life Cycle Assessments (LCA) and the treatment of by-products from the food industry are gaining importance because of the environmental concern. In this context, using sucrose and/or lactose arising from different by-products or underutilized materials has acquired great importance. As sucrose and lactose are highly available in such kind of products, different attempts have been carried out to use them as raw materials for the synthesis of FOS and GOS, thus adding value to these underutilized products.

Some examples of products available for the synthesis of FOS include carob flour, containing *ca*. 50% sucrose, which has been used as substrate with similar yields than pure sucrose in equivalent concentrations (222). The use of grape must, mainly composed of glucose and fructose, for the synthesis of FOS is a recent and very interesting strategy to add value to a by-product highly available in wine producing countries (223). In addition, sugar syrup and molasses from beet processing containing sucrose were reported to be low-cost and available substrates for the enzymatic synthesis of FOS (54).

In turn, using by-products rich in lactose has been a quite extended strategy for the synthesis of GOS. This is the case of whey permeate. Whey is the by-product remaining from the production of cheese. It is majorly composed of proteins of high biological value (i.e., β-lactoglobulin) and lactose. Whey is generally spray-dried and powders are manufactured as three main products (136): whey protein concentrate, containing 70–85% of the milk whey proteins and 50% of the milk lactose; Whey protein isolate, containing more protein (90–98%) than whey protein concentrates; Whey permeate, essentially composed of lactose and some minerals.

Whey proteins are usually incorporated in the formulation of bakery, meat and dairy products, as well as in infant and sportive food products. The remaining whey permeate is currently used for the production of refined lactose. The obtaining of GOS from whey permeate enables the valorization of whey surplus that economically are not feasible to dry (50). In this regard, milk, sweet and acid whey have also been used as substrate for the synthesis of GOS (149). In addition, different attempts have been used to obtain GOS from whey permeate. Golowczyc et al. (224) used this by-product first to obtain GOS, and then as culture and dehydration medium for probiotic lactobacilli. In turn, Nestle company uses demineralized sweet whey permeate as a food grade source of lactose for the synthesis of GOS. To this aim, the partially demineralized whey permeate containing lactose is evaporated to achieve 50% total dry matter, and then incubated with beta-galactosidases from *A. oryzae* to obtain GOS with DP within 2–5. After synthesis, the enzyme is denatured and inactivated by heating, and the products, containing GOS, mono and disaccharides, purified by membrane nanofiltration, and finally dehydrated.

## Properties and Applications

As mentioned before, the main characteristic of FOS and GOS is their prebiotic effect: both of them are non-digestible food ingredients that selectively stimulate the growth and/or activity of potentially health-enhancing intestinal bacteria (6). Short chain FOS and GOS (DP <5) were especially recognized to encourage the growth of beneficial bacteria in the colon. They act as fermentative substrates, and undergo fermentation in the colon of the host (42, 114). This capacity discourages the growth of potential pathogens in the colon, enhancing the defense mechanisms of the host and protecting against enteric infections. Additionally, this increases mineral absorption and immunomodulation for the prevention of allergies and gut inflammatory conditions; furthermore, they are being investigated as possible reducers of risk factors for colon cancer (4, 42).

Strongly related to the non-digestible characteristic, FOS and GOS are identified as dietary fiber. The European regulation on food labeling obliges the manufacturers to identify these ingredients as dietary fiber (42, 114). In fact, the recent legal definition of fiber is “carbohydrate polymers with 3 or more monomeric units, which are neither digested nor absorbed in the human small intestine obtained from food raw material by physical, enzymatic, or chemical means and which have a beneficial physiological effect demonstrated by generally accepted scientific evidence” (225). Among their nutritional properties, GOS and FOS are carbohydrates that reduce basal hepatic glucose production without any effect on insulin stimulated glucose metabolism, which makes them suitable for diabetic diets (226, 227). Furthermore, they affect lipid metabolism control counteracting triglyceride metabolism disorder and reducing free cholesterol level (42, 227).

Beyond their already known nutritional and prebiotic properties, FOS and GOS have technological properties that are strongly determined by their composition. Both inulin and oligofructose are quite stable toward disadvantageous technological conditions, namely low pH, high temperatures and low dry solids conditions. In extreme conditions, FOS and inulin are not hydrolyzed when the pH is above 4.5 and the storage temperature is below 10°C. The greater the degree of polymerization, the more stable the oligosaccharide. On this basis, they were used in a wide spectrum of technological applications either as syrups or as powders.

The (2→ 1) glycosidic bonds of inulin make it indigestible to humans and it can therefore be used as a low-calorie sweetener, fat replacer and dietary fiber (228). Short chain FOS are those used for sugar reduction. The technical properties of oligofructose, such as solubility, taste and viscosity, make it a suitable ingredient to reduce the sugar content and increase the fiber content of many food products (i.e., jams, candies, gums, marshmallows) without affecting their organoleptic properties (42, 117, 122). FOS and inulin have been successfully incorporated as sugar replacers in the formulation of dairy products (mainly in yogurts) following the concept “sugar out, fiber in” and “fat out, fiber in.” Bakery products, including bread, cookies, cakes and muffins, are other group of products that have benefited from the addition of FOS and inulin in replacement of sugar. Cereals (i.e., breakfast cereals, cereal bars) represent another food category that suits the “sugar out, fiber in” concept, and in which oligofructose has been adequately used in replacement of part of the sugars, leading to products resembling the sugar texture very closely. For example, due to its excellent binding properties and good moisture retention, oligofructose is currently used as a binder of granola bars, leading to an improvement of their shelf-life (oligofructose acts as a humectant, inhibiting the hardening during storage).

Inulin is able to form gels, whose rheological properties are directly related with their crystallization behavior. The primary non-spherical inulin crystallites combine to more or less spherical aggregates which interact to form a weak structured gel where a significant amount of water is immobilized. When inulin is incorporated in a food product the formation of these crystalline aggregates results in an enhanced creaminess and mouthfeel even at dosages much lower than those needed for gel formation (42, 70, 229). These properties make them excellent textures modifiers. Indeed, the addition of inulin to a low fat food product improves his creaminess and texture. The fat replacement and texturizing properties are related to the particle gel behavior. Hence, inulin is an excellent fat-replacer for water containing food systems, where inulin is present as small particles mimicking the mouthfeel and mouthcoating properties of fat. After shearing, inulin particles are formed with a size between 1 and 3 μm which is also the size of fat droplets after homogenization. This property enables the reduction of the caloric content of many products, including dairy products (yogurts, dairy desserts, custards, ice-creams), bakery (cake systems, puff-pastry, croissants, scones). Another category of foods benefiting from the fat-replacement properties of inulin are emulsified meat products, sauces, prepared meals, meal replacers, sausages and pates, which can be obtained with a creamier and juicier mouthfeel and improved stability thanks to the better water immobilization when replacing fat with inulin. Finally, the solubility of inulin and FOS makes them suitable to enrich beverages (dairy beverages, dairy analogs based on soy, rice, almonds or oat, near waters, fruit beverages), converting them in fiber enriched ones.

As a whole, inulin and FOS are natural ingredients highly versatile, whose applications are beyond their functional properties, making them very attractive in the food industry. The combination of the nutritional properties of fiber with the possibility to reduce sugar and fat give fructans a unique position in the ingredient world.

Regarding GOS, Japanese companies were pioneers in introducing them to the market, during the 1990s. At present, most of the applications of GOS are associated to their incorporation into infant products, with the aim of formulating products that more closely approximated human milk. Although their incorporation into food products is clearly regulated in the legislations of USA, European Union, Australia, New Zealand, Argentina and Brazil, their incorporation into other food products is rather limited in comparison to that of FOS. In this regard, in Austria, Finland, Italy, Belgium, the Netherlands and Japan, GOS are used as food ingredients in the formulation of dairy products, fruit juices, bread and bakery products, meal replacers, fermented and flavored milks, and cereal bars. Food for elderly and hospitalized people and poultry, pig and aquaculture products are among other applications of GOS as ingredients (114).

Similar to FOS, the composition of GOS determines their physico-chemical properties as food ingredients. GOS are usually commercialized as mixtures of oligosaccharides (>55%), lactose (<20%), glucose (<20%), and a small amount of galactose, in powder or high concentrated syrups. As GOS have the capacity of remaining stable at high temperature treatments (up to 160°C) and at low pH (2–3) (117), they are considered more stable than FOS (230). The shelf-life of GOS exceeds 18 months without microbial spoilage. GOS containing monosaccharides have relatively low Tg (*ca*. 50°C), thus making them very difficult for spray-drying processes. To counterbalance this disadvantage, the use of whey protein concentrates or maltodextrins has been reported (231). In spite of that, mono and disaccharides present in the matrices make the products highly hydroscopic, so that, they must be stored under dry conditions. This hygroscopic character (that is, humectant properties) makes them suitable ingredients to prevent the excessive drying of bread and other bakery products, thus providing a better taste and texture.

One of the most important applications of GOS is as ingredients for infant formulas. Basically they are added to mimic human milk oligosaccharides, which are claimed to be responsible for a number of physiological effects that impact on the development of newborns (4, 197, 232–234). Additionally, in the food industry, GOS are used as sweeteners, not only in such formulas, but also in fermented products (as milk products and breads), jams, refreshing water and fruit juices (115). Regarding fermented products, GOS are especially suitable for them because of their stability. For example, during bread making GOS resisted yeast fermentation and baking conditions. What is more, the taste and texture of bread remained preserved (117). In the case of yogurt, GOS besides of being unchanged during the fermentation lactic acid bacteria, studies with consumers suggested that the yogurt with GOS had better sensory attributed (mouthfeel experience) than yogurt without GOS (234). In the case of beverages, particularly fruit juices and soft drinks, GOS are preferred to be incorporated as prebiotic ingredient due to their acid stability and their ability to form clear solutions (213).

Because plant based GOS are not produced from dairy, they are completely lactose free. Growing infant formula demand in China and India as well as application growth in cereals, ice creams and dairy replacement products is expected to have a positive impact on plant based GOS research and development in the near future.

As it was mentioned, besides their application in the food industry, GOS are also relevant in the healthcare industry as constituents in clinical nutrition products (234). These types of products are food and beverages designed for people with a lowered defense system who have specific nutritional needs. These kind of products often contain fiber (both insoluble and soluble) to provide an intestinal function as close as possible to normal food and to prevent constipation or diarrhea. In this sense, from a nutritional point of view, GOS are assumed to be fiber for being non-digestible polysaccharides, so they are suitable for use in different types of medical nutrition concepts, including tube- and sip feed and powdered supplements. Moreover, their stability is extremely important for liquid formulas. In many cases, patients express lactose intolerance. This is why GOS mixtures for this purpose must be lactose-free (213).

GOS prebiotic effect in not limited to human health. They are also interesting ingredients for pet food. They help to maintain animal immune system in right conditions promoting a healthy intestinal environment. Several studies pointed out that GOS consumption favored the generation of lactic acid bacteria such as lactobacilli or bifidobacteria and protected them from pathogens (213). In this line, during the last years, GOS applications in the poultry, pig and aquaculture industries have been rising up. They promote animal's health and growth, improved gut microbial ecology, and reduced diseases, mortality, and fecal odor. Additionally, there was demonstrated that GOS could eliminate methane production by ruminants (114).

## Conclusions

FOS and GOS have been the most investigated compounds with demonstrated prebiotic properties. As they can be obtained either by synthesis or by hydrolysis, they are highly variable in terms of structures. The chirality of GOS obtained by synthesis and by extraction/hydrolysis is opposed (β- in synthesis and α- from extraction/hydrolysis) even though the linkage is identical. Both types of GOS have relevant prebiotic effects. Hence, research work focused on understanding the relationship structure-functionality contributes to the development of the functional food market toward specific health needs.

FOS and GOS are complex structures containing mixtures of oligosaccharides with different degrees of polymerization. Their technological properties strongly depend on their composition which in turn, is a result of the obtainment process. For this reason, an accurate engineering of their production is of great importance to achieve the desired properties. Such engineering depends on many factors, not only technological but also economical. In this regard, the synthesis of FOS and β-GOS has a very important advantage, as substrates (sucrose and lactose) are cost-effective and the reactions can be standardized as there is no variability on the substrates. On the contrary, the natural variability of the raw materials normally used to obtain FOS and α-GOS by hydrolysis can eventually lead to difficulties to standardize the production. However, as α-GOS are assuredly lactose free, their commercial production can be important for relevant market sectors. Standardizing FOS and GOS production by enzymatic synthesis requires the control of the combined effect of reaction conditions (temperature, pH, time, and substrate concentration), enzyme source and activity on the process yield and product composition. In this line, as enzymes are the most expensive input for an economically feasible process, the selected ones are not specific and thus, the reaction conditions must be optimized to achieve a maximum productivity and yield of FOS and GOS. The improvements in immobilization technologies have certainly contributed to overcome this problem in the last years.

Taking into account the advantages and disadvantages of both hydrolysis and synthesis processes, and also the technological properties of the obtained products, an adequate engineering of the processes appears as an important strategy to make the production of FOS and GOS an economically feasible industrial process. This viewpoint is of special interest for small and medium companies, considering the high turnover of FOS and GOS production, which makes the investment in the prebiotic market a very profitable activity.

## Author Contributions

MU, ET, and AG-Z wrote the issues related to the synthesis of GS and FS, as well as the applications and conclusions. GM and PC wrote the issues related to the obtaining of FS and GS by hydrolysis.

### Conflict of Interest Statement

The authors declare that the research was conducted in the absence of any commercial or financial relationships that could be construed as a potential conflict of interest. The reviewer SC declared a shared affiliation, with no collaboration, with several of the authors, MU and AG-Z, to the handling editor at time of review.
